# PAK1 (p21-Activated Kinase 1) and Its Role in Neurodevelopmental Disorders—New Case Report and a Comprehensive Review

**DOI:** 10.3390/ijms27010439

**Published:** 2025-12-31

**Authors:** Natasza Blek, Mikołaj Pielas, Volodymyr Kharytonov, Karolina Rutkowska, Joanna Rusecka, Sławomir Lewicki, Rafał Płoski, Piotr Zwoliński

**Affiliations:** 1 Institute of Clinical Sciences, Maria Sklodowska-Curie Medical Academy in Warsaw, Pl. Żelaznej Bramy 10, 00-136 Warsaw, Poland; 2 Neurosphera Epilepsy, Neurology and Psychiatry Centre, 02-829 Warsaw, Poland; mikolaj.pielas@neurosphera.pl (M.P.);; 3 Department of Neurology and Epileptology, The Children’s Memorial Health Institute, 04-730 Warsaw, Poland; 4 Clinical Hospital “Psychiatry”, 04080 Kyiv, Ukraine; 5 Department of Medical Genetics, Medical University of Warsaw, 02-106 Warsaw, Poland; karolina.rutkowska@wum.edu.pl (K.R.);; 6 Institute of Outcomes Research, Maria Sklodowska-Curie Medical Academy in Warsaw, Pl. Żelaznej Bramy 10, 00-136 Warsaw, Polandslawomir.lewicki@uczelniamedyczna.com.pl (S.L.)

**Keywords:** PAK1, neurodevelopmental disorder, de novo variant, genotype-phenotype correlation

## Abstract

Pathogenic variants in the *PAK1* gene are linked to neurodevelopmental and neurodegenerative disorders by disrupting neuronal signaling and function. Despite increasing recognition, the mechanisms underlying these conditions remain incompletely understood, limiting therapeutic options. Here, we report a novel de novo *PAK1* variant, c.396C>A (p.Asn132Lys), in a 5-year-old girl with Intellectual Developmental Disorder with Macrocephaly, Seizures, and Speech Delay (IDDMSSD). The patient presented with mild intellectual disability, delayed speech, macrocephaly, hypotonia, gait ataxia, autism-like behaviors, and focal epileptiform activity. Trio exome sequencing confirmed the variant as likely pathogenic, absent in her parents and population databases. This finding expands the phenotypic spectrum of PAK1-related disorders and underscores the critical role of the autoinhibitory domain in neurodevelopment. In addition, we performed a comprehensive literature review of *PAK1* variants affecting both the autoregulatory and kinase domains, summarizing associated clinical features and pathogenic mechanisms. Our study highlights the importance of identifying *PAK1* pathogenic variants for accurate diagnosis, refined genotype-phenotype correlations, and the development of potential targeted therapeutic strategies. By integrating novel case data with existing literature, this work advances understanding of *PAK1*-related neurodevelopmental disorders and supports the application of genetic analysis in rare pediatric NDD cases.

## 1. Introduction

Neurodevelopmental disorders (NDDs) comprise a heterogeneous group of conditions affecting brain development and function, leading to functional deficits of the central nervous system and clinical manifestations in childhood or adulthood. As a consequence, it leads to disorders such as intellectual disability, developmental delay, autism spectrum disorders, and developmental motor abnormalities. All conditions are associated with significant limitations in intellectual functioning and adaptive behaviors that reflect an impairment in personal, family, social, educational, occupational, or other important areas of functioning [1,2]. While diagnostic criteria for NDDs provide guidelines for professionals, it is still difficult because some symptoms may be interpreted differently or subjectively by individual physicians. NDDs are diverse in etiology, onset, and severity.

Patients with NDDs often demonstrate additional neurological (e.g., seizures, hypotonia) or non-neurological features (e.g., dysmorphic features, congenital abnormalities, cardiac involvement). Clinical manifestations and their severity, even within the same family, are variable. This complex phenotype makes it difficult to diagnose, as it can be challenging to differentiate between main and secondary symptoms.

The genetic background of NDDs is also complex. NDDs may be caused by a mutation in one gene, e.g., *TBR1* (MIM *604616) correlated with intellectual developmental disorder with autism and speech delay (MIM #606053), or may concern multiple genes, leading to chromosomal aberrations, e.g., 7q11.23 duplication, typically associated with speech and language delay, motor delay, intellectual disability, anxiety, autism, or 16p11.2 deletion—common features include developmental delay, speech impairment, intellectual disability, or autism, behavioral problems, hypotonia, seizures, and increased weight gain [3]). Variants can change the gene sequence (e.g., missense, nonsense, frameshift, small insertion, or deletion) or can even change the number of copies of some genes (copy number variation, CNV). They may be inherited in all manners (autosomal dominant, recessive, or X-linked). Diagnosis of NDDs may be difficult, also due to incomplete penetrance and variable expressivity, which are characteristic of many defects in genes involved in psychomotor development. Moreover, genetic and molecular analysis is made difficult due to the small number of affected patients. Up to 40% of NDDs have a documented underlying monogenic defect, primarily due to de novo variants [4]. Moreover, DNA methylation, histone modifications/variants, ATP-dependent chromatin remodeling, as well as regulatory non-coding RNAs, regulate diverse aspects of neuronal development, and alterations in epigenomic marks have been associated with NDDs of varying phenotypes [5]. In summary, diagnosing NDDs requires a careful, holistic approach that considers symptoms, developmental milestones, family history, and environmental factors.

Neurodevelopmental disorders are a major public health concern, as they encompass a range of conditions affecting the development of the brain and nervous system. Prevalence rates can vary depending on the specific disorder and population studied. It is estimated that between 1 and 5% of the population could have some form of neurodevelopmental disorder. A 2019 study by the World Health Organization (WHO) estimated that globally, about 1 in 7 children (14%) suffer from at least one neurodevelopmental disorder (this group includes autism spectrum disorder-ASD, intellectual and developmental disabilities-IDD, attention deficit hyperactivity disorder-ADHD, learning disabilities, and developmental coordination disorders). The prevalence of ASD has been rising, and recent studies estimate that it affects 1 in 36 children in the United States [6].

Here, we firstly present the effect of the novel *PAK1* de novo variant, which caused Intellectual Developmental Disorder with Macrocephaly, Seizures, and Speech Delay. The patient showed mild intellectual disability, speech delay, macrocephaly, hypotonia, gait ataxia, autism-like behaviors, and focal epileptiform activity.

## 2. Detailed Case Description

### 2.1. Patient

The subject presented is a Caucasian 5-year-old girl, born at 39 weeks’ gestational age to non-consanguineous parents with no significant medical history. The mother was G3P1 (three pregnancies, one live birth). Due to two early pregnancy failures, the karyotypes of the mother and father were performed, which did not reveal any pathology. No pregnancy and birth complications were seen, with a body weight of 3850 g and a 10/10 Apgar score.

### 2.2. DNA Analysis

The proband’s and her parents’ DNA were extracted from peripheral blood. Then, TRIO Exome Sequencing (TRIO ES) using the Twist Human Core Exome, Twist mtDNA Panel, Twist RefSeq Panel, and ClinVar Custom Panel (Twist Bioscience, South San Francisco, CA, USA) was performed according to the manufacturer’s instructions. Paired-end sequencing (2 × 100 bp) of the enriched libraries was conducted on the NovaSeq 6000 platform (Illumina, San Diego, CA, USA). The mean coverage of the proband’s sample was 115 (99.5% of the target was covered ≥20× and 99.5% of the target was covered ≥10×). For the mother’s sample, the mean coverage was 129 (99.5% of the target was covered ≥20×, 99.5% of bases had coverage ≥10×); for the father’s sample, the respective values were 127, 99.6%, and 99.7%. The raw ES data underwent bioinformatic analysis, and variant prioritization was performed. The variants were called using the following variant callers: HaplotypeCaller, DeepVariant, FreeBayes, and Mutect2.

### 2.3. Functional Analysis

The identified variants were annotated with functional details and population frequency information, including data from the gnomAD database (http://gnomad.broadinstitute.org/, accessed on 24 April 2024) and an in-house database of >8500 Polish individuals. The variant annotation involved both the ClinVar (www.ncbi.nlm.nih.gov/clinvar/, accessed on 24 April 2024) and HGMD (www.hgmd.cf.ac.uk, accessed on 24 April 2024) databases. Then, in silico pathogenicity variant prediction was carried out utilizing the in-house developed platform GeneBe (https://genebe.net/, accessed on 24 April 2024) and data provided by Varsome [7,8]. The analysis also included the guidelines set by the American College of Medical Genetics and Genomics (ACMG) [9].

For the detection of large CNVs (copy number variants) the CNVkit was used [10]. Each sample was compared to a precomputed reference built from approximately 50 samples prepared using the same enrichment protocol. For the analysis of smaller CNVs, typically spanning a few exons, DECoN was used [11]. In this case, the reference set consists of samples prepared within the same library and sequenced in the same experiment.

### 2.4. Clinical and Neurological Evaluation of the Patient

Regarding the child’s gross motor skills, all the milestones were reached within the upper age limit. The proband is ambulatory but requires parental supervision due to mild gait ataxia and motor hyperactivity. Her speech development is delayed, but she remains verbal, with the ability to construct sentences of 2–3 words. The neuropsychology evaluation (Stanford-Binet 5) was performed, and it revealed mild intellectual disability.

On physical examination, generalized hypotonia, macrocephaly (head circumference 53 cm, +2.2 SD), and a wide sandal gap were reported. Other findings included sleep disturbances, autism-like behavior, attention deficits, myopia, convergent strabismus, and neck and lumbar hemangiomas.

**Figure 1:**
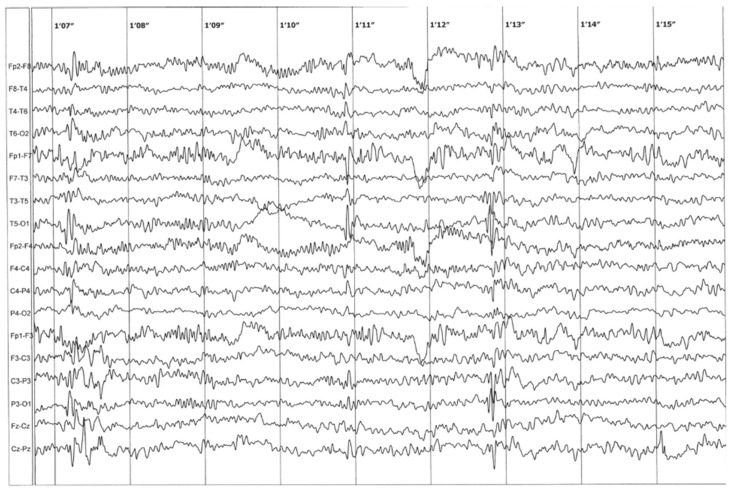
Awake electroencephalographic study, 10–20 system electrode placement: paroxysmal focal epileptiform activity, spikes of high amplitude, and slow wave complexes with maximum amplitude in the occipital regions.

At the age of 2, she developed her first seizures with tonic–clonic and myoclonic semiology. Electroencephalographic studies were performed, and the results showed the presence of paroxysmal focal epileptiform activity, spikes of high amplitude (350 μV), and slow wave complexes localized in the occipital regions, predominantly on the left, with occasional secondary bilateral synchronization (Figure 1). A magnetic resonance imaging (MRI) study revealed a single choroid plexus cyst with no other pathologies.

### 2.5. Results of DNA Analysis

**Figure 2:**
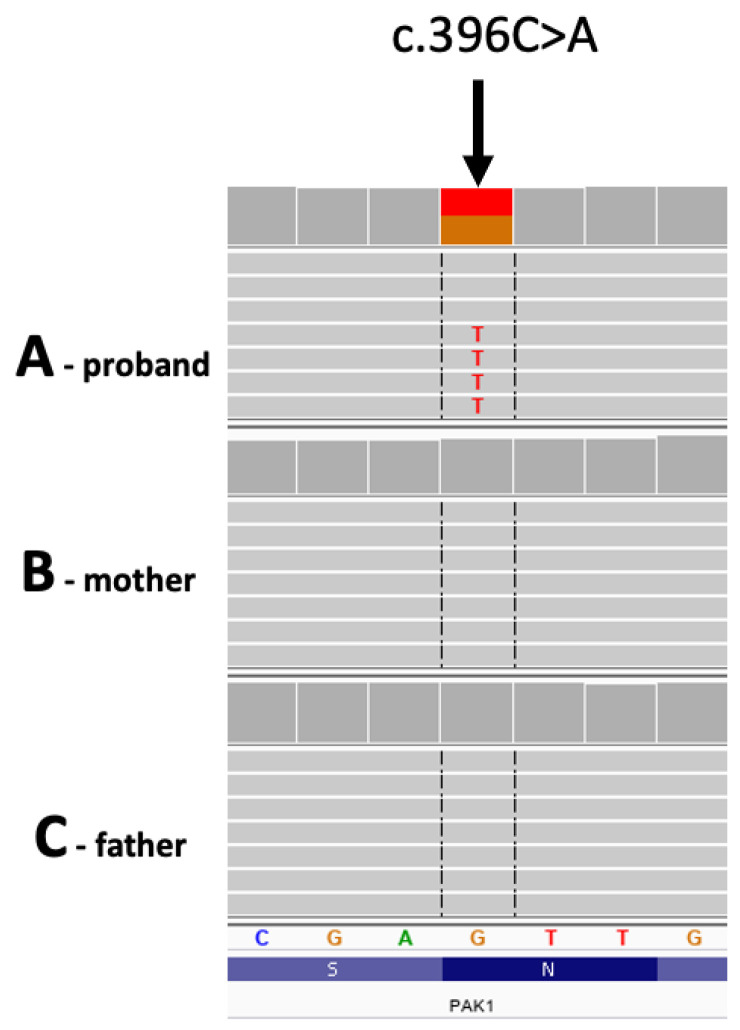
IGV visualization of the identified de novo c.396C>A (NM_002576) variant in the *PAK1* gene, found in a patient diagnosed with Intellectual Developmental Disorder with Macrocephaly, Seizures, and Speech Delay (IDDMSSD). (**A**) Proband; (**B**) mother; (**C**) father.

TRIO ES analysis revealed a novel missense heterozygous c.396C>A variant in the *PAK1* gene (GRCh38/hg38: 11:077379284-G>T, NM_002576.5, p.(Asn132Lys)). The variant was not found in the proband’s parents, probably indicating a de novo event (Figure 2). The quality of the variant (Phred quality score) was 766, with 24 and 11 reads from either strand. The total coverage of the variant was 69 reads. The c.396C>A variant was absent in the control gnomAD v4 database and in the in-house database of ES of Polish individuals. The in silico AlphaMissense tool predicts the pathogenic outcome of the variant [12]. According to the ACMG guidelines, the c.396C>A variant was classified as likely pathogenic (6 points: PM1 moderate, PM2 moderate, PM6 moderate, PP2 supporting, BP4 supporting). The c.396C>A variant in the PAK1 gene was likely located within a hotspot region. Across the whole PAK1 gene (546 amino acids according to the NM_002576.5), there were 2 pathogenic and 23 likely pathogenic variants. The c.396C>A variant was located around another 11 pathogenic/likely pathogenic variants, as illustrated in the figure in Section 3.4.2. This supported the application of the PM1 criterion. No other SNV variants potentially causing NDDs were identified in our patient. The analysis of CNV showed no changes that could explain the proband’s phenotype.

According to the Online Mendelian Inheritance in Man (OMIM; https://omim.org/about, accessed on 24 April 2024), pathogenic variants of the *PAK1* gene (so far only missense variants) were described in association with inTellectual Developmental Disorder with Macrocephaly, Seizures, and Speech Delay in an autosomal dominant mode of inheritance (MIM #618158).

Sanger sequencing was performed on the ABI3730 sequencer. Sequences were visually inspected on the FinchTV chromatogram viewer. Primer sequences are listed below: (forward primer: 5′-AAACGTGTGCAGTGACAGAGTGAAG-3′; reverse primer: 5′-GCATCTTTTGCTGCTAGCAAGTGTC-3′).

**Figure 3:**
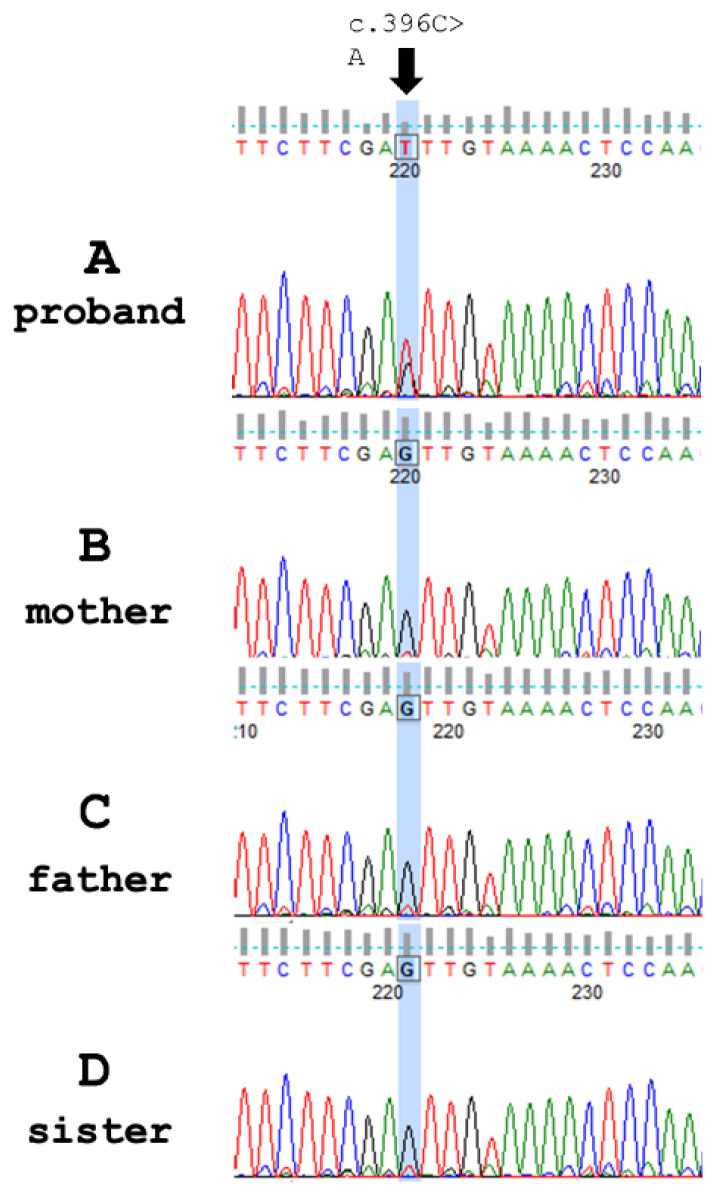
Electropherograms of the genomic DNA region (gDNA) harboring the PAK1 variant obtained by Sanger sequencing in the (**A**) proband, (**B**) mother, (**C**) father, and (**D**) sister.

We confirmed the presence of the c.396C>A variant in the proband as well as its likely de novo origin (Figure 3).

In search of other disease causes, we analyzed de novo, homozygous, and compound heterozygous variants in our patient (we considered variants with frequency below 1% in gnomAD and an in-house database of >8500 exomes, as well as variants labeled as pathogenic or likely pathogenic in the ClinVar database, irrespective of frequency). Given the clinical status (healthy parents affected female child), we considered autosomal recessive inheritance (homozygous or compound heterozygous variants) or dominant inheritance (de novo variants). Apart from the PAK1 variant, we did not identify additional plausible de novo and homozygous variants in genes associated with human disease listed in the OMIM (https://omim.org/, accessed on 17 December 2025) and GenCC (https://search.thegencc.org/, accessed on 17 December 2025) databases.

Regarding compound heterozygous variants, we identified two changes in the NBEAL2 gene: a synonymous c.5769G>A variant (GRCh38/hg38: 3:47003864-G>A) inherited from the father with a gnomAD allele frequency of 0.0005455, and a missense c.4810C>T (GRCh38/hg38: 3:47001947-C>T) variant inherited from the mother, which was absent from gnomAD. According to GeneBe (https://genebe.net/, accessed on 17 December 2025), the identified variants received −7 ACMG points for the c.5769G>A and 3 ACMG points for the c.4810C>T variant. The NBEAL2 gene has been described in OMIM as being associated with gray platelet syndrome with an autosomal recessive mode of inheritance (MIM #139090). Since this phenotype did not correlate with the proband’s clinical features, the variants were excluded as disease-causing.

Regarding de novo variants, we identified the c.1023C>A variant in the ADH7 gene (GRCh38/hg38: 4:99415555-G>T), which was absent from both gnomAD and the in-house database. In silico tools predicted a benign effect for this variant (for example, AlphaMissense score: 0.22; CADD score: 12; REVEL score: 0.076). The c.1023C>A variant in the ADH7; no clinical diagnostic laboratories have submitted clinical significance assessments for this variant to ClinVar and received 0 ACMG points in GeneBe. At the same chromosomal position, two additional variants have been identified: a missense c.1023C>G (GRCh38/hg38: 4:99415555-G>C) variant (present in one individual in gnomAD) and a synonymous c.1023C>T (GRCh38/hg38: 4:99415555-G>A) variant (absent in gnomAD, present in 4 persons from the Regeneron database—https://rgc-research.regeneron.com/, accessed on 17 December 2025). In Genebe, the variants received 0 ACMG points for c.1023C>G and -3 ACMG points for c.1023C>T. Both variants have not been reported in the ClinVar database and were predicted as benign by in silico tools. According to OMIM, the ADH7 gene encodes a class IV alcohol dehydrogenase (MIM *600086). The ADH7 gene has not been associated with human disease in the OMIM or GenCC databases. The probability of tolerance to both heterozygous and homozygous loss-of-function (pNULL) variants in the ADH7 gene was relatively high (0.8). On the basis of all the arguments mentioned above, the c.1023C>A variant in the ADH7 gene has not been considered as being associated with the proband phenotype.

## 3. Discussion

To the best of our knowledge, this is one of the few reported cases of *PAK1* variants causing Intellectual Developmental Disorder with Macrocephaly, Seizures, and Speech Delay. Our findings align with previous reports of de novo *PAK1* variants leading to similar phenotypic presentations, including macrocephaly, seizures, and intellectual disability. Identifying this novel variant further expands the phenotypic spectrum of IDDMSSD and underscores the importance of considering *PAK1* pathogenic variants in patients with similar clinical features. Improved understanding of the clinical manifestations associated with specific *PAK1* variants will enhance diagnostic accuracy, inform prognosis, and potentially guide targeted therapeutic strategies.

Due to the limited number of studies in this area and to better understand the role of the pathogenic *PAK1* gene variant described in this study in Intellectual Developmental Disorder with Macrocephaly, Seizures, and Speech Delay (IDDMSSD), we aimed to present below the physiological role of PAK1 kinase in the organism, as well as to discuss the pathogenic variants occurring within the two domains of this protein, the autoinhibitory domain and the kinase domain, in the context of pathogenesis.

### 3.1. PAK1 Structure

PAK1 kinase is encoded by the *PAK1* gene (HGNC:8590) in chromosome 11 (11q13.5-q14.1). The gene contains 23 exons (NM_002576.5), of which six exons are for 5′-UTR, and seventeen encode protein [13]. Alternative splicing results in multiple transcript variants [14]. PAKs are classified into two groups, A (PAK1–PAK3) and B (PAK4–PAK6), based on their sequence identity, structure, and regulatory mechanisms [13]. They are implicated in various types of human disorders. Group A PAKs display broad low to moderate expression across human tissues with relatively higher transcript levels in the nervous system [15,16,17].

PAK1 is a serine/threonine kinase that plays crucial roles in various cellular processes [13,14]. As a key downstream effector of RAC1 and CDC42 small GTPases, PAK1 regulates cytoskeletal dynamics, cell motility, and signal transduction pathways essential for proper nervous system development [15,16]. Human PAK1 (NP_002567.3) is a 545-amino acid protein (65 kDa). Structurally, human PAK1 exhibits a modular organization defined by two major functional domains: the Cdc42/Rac interactive binding (CRIB) domain and the C-terminal protein kinase domain. The N-terminal autoregulatory region-PBD/AID (protein binding domain/autoinhibitory domain; 70–140 aa) contains the CRIB domain, spanning amino acids 75–88. This region lies within a broader GTPase-binding segment (75–105). Surrounding these elements is the autoregulatory region (ARR), located between residues 70 and 140, which contributes to maintaining the kinase in an inhibited conformation. The C-terminal part of PAK1 comprises the protein kinase domain, extending from amino acids 270–521. This domain adopts the characteristic bilobal architecture typical of serine/threonine kinases and contains the catalytic motifs required for phosphorylation. Together, these regions define the modular structural organization of PAK1, separating its regulatory N-terminal elements from its C-terminal catalytic core [14,17,18,19].

PAK1 exists as an autoinhibited homodimer, in which the regulatory domain (AID) suppresses the kinase domain responsible for the catalytic activity of PAK1. Activation occurs upon binding of GTP-loaded Cdc42 or Rac1 to the protein-binding domain (PBD), leading to Thr423 phosphorylation, dimer dissociation, conformation changes, and kinase activation through autophosphorylation [20]. Upon activation, the kinase phosphorylates a range of downstream substrates involved in actin cytoskeleton remodeling, apoptosis, and cell cycle regulation. The kinase domain utilizes ATP to transfer phosphate groups to serine/threonine residues on target proteins. The ATP-binding site within this domain facilitates the transfer of the phosphate group. The C-terminal domain (which includes the PBD and CRIB motif) is involved in both GTPase binding and interactions with other signaling proteins. The CRIB motif is a sequence that specifically interacts with the GTP-bound forms of small GTPases Cdc42 and Rac1.

In addition to PBD binding sites, kinase PAK1 contains additional serine/threonine phosphorylation sites that can undergo phosphorylation [21]. These sites are key for modulating the function of PAK1 itself, as well as for regulating its interactions with other proteins. The sites are generally divided into three groups: 1. Autophosphorylation—PAK1 phosphorylates itself to regulate activation and substrate interactions; 2. Phosphorylation sites modified by other kinases; and 3. Sites phosphorylated by both PAK1 and external kinases [22]. Phosphorylation of PAK1 can promote its activation or alter its interaction with substrates and scaffolding proteins. Phosphorylation at certain serine/threonine residues in the activation loop of the kinase domain is necessary for PAK1 to adopt an active conformation. Other phosphorylation sites regulate interactions with downstream effectors and regulatory proteins. Phosphorylation controls actin dynamics, cell movement, and survival [20].

### 3.2. PAK1 Functions

PAK1 is a ubiquitous kinase that regulates various cellular processes, particularly cytoskeletal organization, signal transduction, and cell growth. It also plays essential roles in migration, differentiation, apoptosis, and energy metabolism.

PAK1 plays a pivotal role in remodeling the actin cytoskeleton, which is crucial for cellular movement and shape. It phosphorylates cofilin, a protein that promotes the severing of actin filaments, facilitating actin turnover and lamellipodia formation at the leading edge of migrating cells [23]. PAK1 also phosphorylates paxillin, an adaptor protein that links integrins to the actin cytoskeleton, promoting cell adhesion and migration [24]. Another protein activated by PAK1 is filamin A, which modulates actin filaments and facilitates the attachment of various proteins to the cytoskeleton. This interaction plays a crucial role in regulating cell adhesion and migration [21].

PAK1 plays a crucial role in regulating cell proliferation and apoptosis. During the cell cycle, PAK1 mediates the phosphorylation of multiple target proteins, including NFκB, CRAF, Aurora A, ARPC1b, PLK1, histone H3, and MORC2 [25]. Additionally, PAK1 is involved in the modulation of cyclin and cyclin-dependent kinase (CDK) family activity and expression [26], as well as the activation of retinoblastoma (Rb) and E2F transcription factors [27]. It also promotes survival of the cells by participating in the regulation of the PI3K-PDK1-AKT-mTOR pathway [28].

Due to its role in promoting cell survival and proliferation, PAK1 kinase is frequently overexpressed in cancer cells. Elevated PAK1 levels have been associated with various malignancies, including breast, colon, ovarian, hepatic, pancreatic, glioma, colorectal, prostate, and lung cancers [29] or T-cell lymphoma [30]. Moreover, PAK1 inhibits the apoptotic process by regulating BAD phosphorylation [31] and, together with fibroblast growth factor (bFGF) and vascular endothelial growth factor (VEGF)-mediated Raf-1 and MEK1 activation, promotes cell survival and angiogenesis [32]. The pro-tumorigenic properties of PAK1, along with its high expression in tumor cells, have established this protein as a persistent therapeutic target in cancer treatment. Inhibition of PAK1 activity has been widely described to significantly reduce cell proliferation, promote apoptosis in various cancer cell types, and reduce tumor size and extend the lifespan of treated animals [33,34,35]. The role of PAK1 kinase can also be considered at the tissue/organ level. This kinase plays an important role in heart [36,37], liver disorders [38,39,40], neurodevelopmental disturbances [41], and immune system function [42,43,44].

### 3.3. PAK1 Kinase in Neurodevelopment

One of the recent findings that may be underlying causes of NDDs is abnormalities of serine/threonine p21-activating kinases (PAKs). It regulates the development of dendritic spines, which are essential for synaptic function and learning [45]. PAK1 is crucial for neuronal development, particularly in neurogenesis, dendritic spine formation, and synaptic plasticity. During neurodevelopment, PAK1 coordinates multiple essential processes through its regulation of the actin cytoskeleton and various signaling cascades [46,47]. In neuronal migration, PAK1 controls cytoskeletal rearrangements required for proper cortical development by phosphorylating targets like LIMK1 and tubulin cofactor B [48,49]. PAK1 also guides neurite outgrowth and axon pathfinding by modulating growth cone dynamics and regulating the balance between actin polymerization and depolymerization [50]. At synapses, PAK1 influences dendritic spine morphogenesis and synaptic plasticity by controlling actin dynamics and AMPA receptor trafficking [51,52]. Additionally, PAK1 promotes neural progenitor proliferation and differentiation during neurogenesis [53].

PAK1 is not only highly expressed during embryogenesis but also in adult brain tissue, where it plays a vital role in cell signaling, synaptic properties, and neuronal pathophysiology [54,55,56]. In normal neurons, PAK1 dimers reside in a trans-inhibited conformation, where each autoinhibitory domain covers the kinase domain of the other monomer [57]. In response to synaptic activity, PAK1 plays a role in the regulation of NMDA receptors (important for synaptic plasticity and memory), as well as synapse formation and remodeling. Moreover, PAK1 is a potent regulator of GABAergic synaptic transmission, and its disruption leads to reduced GABA presynaptic release [58].

Pathogenic variants in the PAK1 gene have a significant impact on neurological development, particularly affecting synaptic plasticity, neuronal migration, and cognitive function. These variants often lead to NDDs such as autism spectrum disorder, intellectual disabilities, speech delays, and sometimes seizures. The severity and type of neurological symptoms depend on the nature of the variant, with gain-of-function variants often resulting in more pronounced behavioral and cognitive deficits and loss-of-function variants leading to severe developmental delays and motor impairments. Missense mutations occurring in the PBD/AID region of human *PAK1* are consistently interpreted as gain-of-function variants. Published studies show that these mutations disrupt the normal autoinhibitory interaction between the regulatory N-terminal region and the kinase domain or impair inhibitory dimer formation. As a result, they lead to partial or constitutive activation of the kinase [14,59].

More information about the exact mechanistic effects of specific mutations will emerge, aiding in the development of potential therapeutic strategies for affected individuals. The spatiotemporal regulation of PAK1 activity, which is essential for normal neurodevelopment, can also be disrupted by pathogenic variants [60]. Changes in substrate specificity may cause aberrant phosphorylation patterns, while some variants may exert dominant-negative effects by interfering with wild-type PAK1 function [61,62].

### 3.4. PAK1-Related Neurodegenerative Disorders

In recent years, tremendous progress in the development and availability of genetic diagnostic tools, especially whole-exome (WES) and genome sequencing (WGS), has led to the discovery of novel genes and genetic variants causing NDDs [63,64]. One of the increasingly used diagnostic approaches is familial WES (e.g., trio, quattro) to analyze de novo genetic variants. Knowledge of influenced genes and molecular pathology mechanisms may contribute to implementing personalized and more efficient therapy strategies [59]. Defects in the *PAK1* gene have been identified as a rare cause of pediatric NDDs. These molecular perturbations manifest clinically as a spectrum of neurodevelopmental phenotypes characterized by intellectual disability, macrocephaly, and seizures [65]. The condition, termed Intellectual Developmental Disorder with Macrocephaly, Seizures, and Speech Delay, shows variable expressivity likely related to the specific nature and location of *PAK1* variants [66,67]. Variants cluster in either the kinase domain or autoregulatory domain, potentially explaining some of the phenotypic differences observed between affected individuals [68]. Understanding the complex mechanisms by which *PAK1* variants lead to NDDs is crucial for developing targeted therapeutic approaches for affected individuals [69].

**Figure 4:**
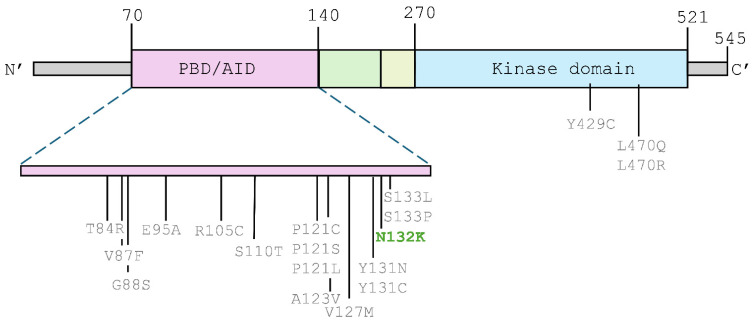
Characteristics of the structure and enzymatic activity of *PAK1* variants. Human PAK1 protein consists of two main domains: the N’ terminal autoregulatory region-PBD/AID (protein binding domain/autoinhibitory domain; 70–140 aa) and the kinase domain (270–521 aa). Pathogenic and likely pathogenic variants described in ClinVar (11 November 2024) and literature are in gray. The novel variant N132K detected in the described patients is highlighted in green. Single-letter amino acid codes were used in the diagram to enhance clarity and readability.

Pathogenic variants in the *PAK1* gene disrupt crucial developmental processes through several mechanisms [69]. Variants affecting either the autoinhibitory or kinase domain can lead to constitutive activation or impaired kinase function, resulting in dysregulated phosphorylation of downstream targets [19,70]. Some variants interfere with protein–protein interactions necessary for proper signaling complex assembly and localization [66]. Here, we present only the effect of gene variants in the autoinhibitory or kinase domain (Figure 4, Table 1)

#### 3.4.1. Variants in Autoinhibitory Domain

The first study to describe the association between *PAK1* variants and IDDMSSD was published by Harms et al. in 2018 [69]. The authors identified a de novo heterozygous missense variant within the autoinhibitory domain of the *PAK1* gene, specifically c.392A>G (p.Tyr131Cys) in a pediatric patient. The affected individual presented with moderate motor developmental delay, moderate receptive language impairment, speech difficulties, and severe intellectual disability, as well as both febrile and afebrile seizures, which were responsive to pharmacological treatment.

Subsequently, Horn et al. in 2019 [19] reported a case involving a c.397T>C (p.Ser133Pro) *PAK1* variant, further supporting the link between *PAK1* variants and NDDs. The subject, a 17-year-old male, exhibited profound developmental delay and intellectual disability, along with autism spectrum disorder. Notably, the patient was non-ambulatory, displayed craniofacial disproportion, and experienced both atonic and tonic–clonic seizures beginning at the age of six.

Also in 2019, Kernohan et al. [70] reported an individual with a novel autosomal dominant NDD characterized by severe regressive autism, intellectual disability, and epilepsy. These clinical features were associated with a variant in the *PAK1* gene, specifically c.362C>T (p.Pro121Leu).

A fourth variant located within the autoinhibitory domain of the *PAK1* gene, c.328T>A (p.Ser110Thr), was reported by Ohoriet al. in 2020 [65]. This variant was associated with an NDD characterized by epilepsy, macrocephaly with focal seizures, absence of verbal speech, and features consistent with autism spectrum disorder.

In 2023, Scorrano et al. [68] described three pediatric patients presenting with severe neurodevelopmental disorders associated with pathogenic variants in the *PAK1* gene. One patient, an 11-year-old child, exhibited moderate intellectual disability, absent expressive speech, and a seizure phenotype that included atypical absence seizures, tonic–clonic, and focal seizures. This clinical presentation was associated with the c.392A>G (p.Tyr131Cys) variant located within the autoinhibitory domain of PAK1. The remaining two patients carried distinct variants at the same genetic locus: c.428T>C (p.Met143Thr) and c.428T>A (p.Met143Lys), which resulted in similar phenotypic features, including severe intellectual disability, profound speech delay, and macrocephaly.

Here, we also report a novel de novo variant in the *PAK1* gene, c.396C>A (p.Asn132Lys), identified in a patient presenting with clinical features of IDDMSSD, which was described before.

In contrast to previously reported *PAK1* variants, our patient did not show MRI evidence of demyelination or delayed myelination, despite the presence of ataxia and seizures. This may indicate phenotypic variability among autoinhibitory domain variants and suggests that the absence of white matter changes could be specific to the p.Asn132Lys variant. However, given the young age at which the MRI was performed, a follow-up study is required to fully assess the degree of myelination.

#### 3.4.2. Functional Impact of Pathogenic Variants in the PAK1 Autoinhibitory Domain

Pathogenic substitutions within the autoinhibitory domain (AID) of PAK1 are predicted to disrupt the regulatory interaction between the AID and the C-terminal kinase domain, which normally stabilizes an autoinhibited dimeric conformation and prevents premature activation. Structural studies have shown that residues between positions 70 and 140, including the CRIB/interacting region, are essential for maintaining this trans-inhibitory interface and regulating Thr423 autophosphorylation [17,57]. Missense variants are reported in the IDDMSSD cluster within this region, and experimental data suggest a gain-of-function mechanism with increased basal kinase activity and enhanced downstream signaling through actin cytoskeleton pathways and synaptic remodeling [19,62,69]. Aberrant activation of PAK1 during neurodevelopment may therefore impair neuronal migration, dendritic spine maturation, and inhibitory/excitatory balance, providing a mechanistic explanation for the convergence of macrocephaly, seizures, and intellectual disability in patients with AID variants. The novel p.Asn132Lys variant identified in our patient lies within this regulatory region, supporting a shared pathogenic model of impaired autoinhibition.

Recent findings in iPSC-derived cerebral organoids from patients with Down syndrome showed that inhibiting the DSCAM–PAK1 pathway restored impaired neurogenesis, demonstrating that excessive PAK1 signaling can be targeted with drugs in a neurodevelopmental setting [71]. Although no specific treatments are currently available for IDDMSSD, these results suggest a possible therapeutic window for variants linked to PAK1 hyperactivation.

#### 3.4.3. Variants in Kinase Domain

To date, three pathogenic variants have been identified within the kinase domain of the *PAK1* gene in association with neurodevelopmental disorders. Two of these variants affect the same nucleotide position, c.1409, resulting in either a c.1409T>A (p.Leu470Gln) or c.1409T>G (p.Leu470Arg) substitution. The c.1409T>A (p.Leu470Gln) variant has been reported to cause profound or severe intellectual disability and absence of verbal communication with macrocephaly and medically refractory seizures [67]. The c.1409T>G (p.Leu470Arg) variant, reported by Horn et al. in 2019 [19], was associated with moderate to severe intellectual disability, non-verbal speech, and exceptionally pronounced macrocephaly (+3.80 SD). Notably, none of the individuals with the *PAK1* variant at the c.1409T position exhibited features of autism spectrum disorder. The last identified pathogenic variant within the kinase domain of PAK1, c.1286A>G (p.Tyr429Cys), was reported by Harms et al. (2018) [69]. The patient exhibited global developmental delay, seizures, hypotonia, and macrocephaly, indicating that this activating mutation in the kinase domain of the PAK1 protein disrupts normal neuronal development and contributes to a neurodegenerative phenotype.

## 4. Conclusions

Pathogenic variants in the *PAK1* gene are associated with neurodevelopmental and neurodegenerative disorders, affecting nervous system function by disturbing cellular signaling and leading to neuronal dysfunction. Despite growing knowledge, understanding of these mechanisms remains limited, which constrains therapeutic approaches and often restricts treatment to symptom management. Here, we have summarized the knowledge about changes in the autoregulatory and kinase domain of the *PAK1* gene that contribute to the development of neurodevelopmental disorders. We have also reported the novel c.396C>A (p.Asn132Lys) variant in a 5-year-old girl with neurodevelopmental disorder, adding important data to the expanding literature on PAK1-related conditions. Identifying such pathogenic variants is essential for improving diagnosis, refining genotype-phenotype correlations, and ultimately supporting the development of more effective therapeutic strategies for neurodevelopmental and neurodegenerative diseases.

## Figures and Tables

**Table 1:** Related neurodegenerative disorders. N/A: Not Available or Not Reported y: Years, m: Months, F: Female, M: Male, SD: Standard Deviation, P: Percentile, WM: White Matter.

	Autoregulatory Domain								Kinase Domain		
Feature	Blek et al.	Patient 1 [69]	Patient 2 [19]	[70]	[65]	Patient 1 [68]	Patient 2 [68]	Patient 3 [68]	[66]	Patient 1 [19]	Patient 2 [69]
Age/Sex	5 y/F	2 y/F	17 y/M	24 y/F	8 y/M	11 y/F	4 y/F	5 y/M	13 y/M	4 y/F	5 y/M
Intellectual Disability	Mild	Severe	Profound	Severe	Severe	Moderate	Severe	Severe	Profound	Moderate to severe	Severe
Speech	Delayed, 2–3 word sentences	Non-verbal	Non-verbal	No meaningful conversation	Non-verbal	Delayed, no active speech	Severe speech delay	Severe speech delay	Non-verbal	Non-verbal	Single words
Macrocephaly	+2.2 SD	+3.96 SD	+2.07 SD	+3.28 SD	+4.93 SD	>P99	Present	Present	Present	+3.80 SD	+4.1 SD
Seizures	Onset 2y, tonic–clonic, myoclonic	Onset 13m	Atonic, tonic–clonic	Generalized tonic–clonic	Focal	Atypical absences, tonic–clonic, focal	Generalized, febrile, infection-triggered	Febrile convulsion, focal seizures	Medically refractory	Onset 19m	Myoclonic, intractable
EEG	Focal epileptiform activity	Abnormal	Paroxysmal discharges	N/A	Sharp waves	Multifocal epileptic discharges	N/A	N/A	Diffuse multifocal epileptic encephalopathy	Abnormal	N/A
MRI Findings	Choroid plexus cyst	Thin corpus callosum, ventriculomegaly	Thin corpus callosum, cerebellar atrophy	Minimal generalized atrophy	Thick corpus callosum	Thick corpus callosum, cerebellar dysplasia	Nonspecific WM signal anomalies	Delayed myelinization	White matter hyperintensities	Thin corpus callosum, ventriculomegaly	White matter hyperintensities
Gait	Mild ataxia	No independent walking	No walking	N/A	Ataxic, unstable	Ataxic	N/A	N/A	Non-ambulatory	Unstable	Ataxic
Hypotonia	Present	Present	Initial hypotonia, later spastic quadriplegia	N/A	Present	Present	Present	Present	Spastic quadriplegia	Present	N/A
Visual Issues	Myopia, convergent strabismus	N/A	N/A	N/A	Strabismus	N/A	N/A	N/A	Cortical blindness	N/A	Strabismus
Sleep Disturbances	Present	N/A	N/A	Present	N/A	N/A	N/A	N/A	Present	N/A	N/A
Other Features	Neck/lumbar hemangiomas, wide sandal gap	N/A	N/A	N/A	N/A	Cerebellar heterotopia	Cervical hydromyelia	N/A	Cleft palate, horseshoe kidney	N/A	Gastroesophageal reflux
PAK1 Variant	c.396C>A (p.Asn132Lys)	c.392A>G (p.Tyr131Cys)	c.397T>C (p.Ser133Pro)	c.362C>T (p.Pro121Leu)	c.328T>A (p.Ser110Thr)	c.392A>G (p.Tyr131Cys)	c.428T>C (p.Met143Thr)	c.428T>A (p.Met143Lys)	c.1409T>A (p.Leu470Gln)	c.1409T>G (p.Leu470Arg)	c.1286A>G (p.Tyr429Cys)

## Data Availability

The original contributions presented in this study are included in the article. Further inquiries can be directed to the corresponding authors.
